# Metabolically Guided Walking and Plant-Based Nutrition Enhance Body Composition and Weight Loss

**DOI:** 10.3390/ijerph23010136

**Published:** 2026-01-22

**Authors:** Harold C. Mayer, Lucas G. Valenca, Gregory W. Heath, Chris S. Hansen, Kristina Nelson Hall, Cassie J. White

**Affiliations:** 1 School of Health and Kinesiology, Southern Adventist University, Collegedale, TN 37315, USA; lucasgvalenca@gmail.com (L.G.V.); cassiewhite@southern.edu (C.J.W.); 2 School of Nursing, Southern Adventist University, Collegedale, TN 37315, USA; knelson@southern.edu; 3 Public Health Program, University of Tennessee at Chattanooga, Chattanooga, TN 37403, USA; gregory-heath@utc.edu; 4 College of Medicine, University of Tennessee Health Science Center, Chattanooga, TN 37403, USA; 5 School of Physics and Engineering, Southern Adventist University, Collegedale, TN 37315, USA; chansen@southern.edu; 6 Institutional Research and Planning, Southern Adventist University, Collegedale, TN 37315, USA

**Keywords:** metabolically guided exercise, respiratory exchange ratio (RER), substrate utilization, moderate-intensity intermittent training, walking intervention, whole-food, plant-based diet, body composition, metabolic flexibility, public health exercise prescription

## Abstract

Sedentary behavior contributes to obesity and metabolic dysfunction, yet few interventions individualize exercise intensity using fuel-based metrics such as the respiratory exchange ratio (RER; VCO_2_/VO_2_). This study investigated the effects of metabolically guided walking combined with whole-food, plant-based nutrition on body composition and metabolic outcomes in sedentary overweight and obese women. Forty-four women mean age 43 years; BMI 30.1 kg·m^−2^) were randomized to low-intensity continuous training (LICT; RER ≈ 0.75), moderate-intensity intermittent training (MIIT; RER ≈ 0.85), or high-intensity continuous training (HICT; RER ≈ 0.95). Following a 2-week dietary lead-in with an individualized ~200 kcal·day^−1^ energy deficit, participants completed an 8-week RER-guided walking program (5 sessions·week^−1^; 15–50 min·session^−1^). Assessments included air-displacement plethysmography (BodPod) body composition, resting metabolic rate and substrate utilization, and oxygen uptake at the first ventilatory threshold (VT1). Data were analyzed using ANCOVA, mixed-factorial ANOVA, and Pearson correlations. Percent body fat decreased significantly across participants (*p* < 0.0001, η^2^ = 0.827), with MIIT demonstrating the most favorable integrated outcomes. MIIT elicited the largest reductions in total body mass (−11.2%), fat mass (−25.9%), and percent body fat (−17.1%), alongside improvements in VT1 VO_2_ (Δ = 1.487 ± 0.895 L·min^−1^; *p* = 0.038). Resting respiratory quotient (RQ) declined in LICT and MIIT but increased in HICT, corresponding with increased fat oxidation in LICT and MIIT and reduced fat oxidation in HICT. Changes in RQ were significantly associated with changes in percent body fat (r = 0.316, *p* = 0.039). Metabolically guided moderate-intensity intermittent walking combined with whole-food, plant-based nutrition produced the most consistent improvements in adiposity, substrate utilization, and submaximal fitness, supporting the public-health feasibility of a community-deliverable, substrate-informed walking prescription.

## 1. Introduction

Sedentary behavior is a major contributor to obesity and cardiometabolic disease, with insufficient physical activity recognized as a leading modifiable risk factor worldwide [1,2]. Despite well-established public health guidelines [3], adherence to structured exercise remains low, particularly among previously sedentary and overweight adults [4]. One contributing factor may be that commonly used standardized exercise intensity prescriptions, while broadly applicable, may not fully reflect individual physiological variability relevant to exercise tolerance and metabolic response [5]

Traditional exercise prescriptions commonly rely on fixed percentages of maximal heart rate or oxygen uptake; however, individuals may exhibit markedly different metabolic responses at equivalent relative intensities [6,7]. Such variability has implications for exercise tolerance, substrate utilization, and long-term adherence, particularly in populations with low fitness or metabolic dysfunction [8]. Consequently, there is growing interest in exercise prescription strategies that incorporate individualized physiological responses to better align training intensity with underlying metabolic demand [8].

Metabolic flexibility, the capacity to appropriately adjust fuel utilization in response to energetic demand—has emerged as an important marker of metabolic health [9]. Impairments in this flexibility are frequently observed in sedentary and obese individuals, suggesting that exercise strategies targeting favorable substrate utilization may hold therapeutic value [9,10,11,12]. Exercise interventions that emphasize tolerable, metabolically appropriate intensities may therefore enhance adherence while supporting favorable cardiometabolic adaptations.

In parallel, dietary patterns emphasizing whole, plant-based foods have been associated with improved body composition and cardiometabolic risk profiles [13,14]. Recent syntheses also suggest that plant-based dietary patterns, including when combined with exercise training, can improve cardiometabolic biomarkers and support aerobic performance without compromising strength outcomes [15,16]. Combining individualized exercise prescriptions with a modest, controlled energy deficit achieved through whole-food, plant-based nutrition may provide a practical and scalable approach for improving metabolic health in sedentary populations.

Accordingly, the purpose of this study was to examine the effects of an eight-week metabolically guided walking program, combined with a whole-food, plant-based dietary intervention, on body composition and body composition, resting metabolism, and submaximal fitness outcomes in sedentary overweight and obese adult women. Participants were assigned to one of three exercise intensity conditions differentiated by metabolic target, allowing comparison of low-, moderate-, and high-intensity metabolically guided walking strategies.

We hypothesized that metabolically guided walking performed at different fuel-utilization intensities, when combined with a whole-food, plant-based diet, would differentially affect body fat, lean body mass, and resting metabolic parameters under equivalent dietary energy restriction.

## 2. Materials and Methods

### 2.1. Ethical Approval and Study Design

This study was approved by the Southern Adventist University Institutional Review Board (22 February 2018). Using a randomized parallel-group design, 44 sedentary overweight or obese women completed a 10-week intervention consisting of a 2-week whole-food, plant-based, non-dairy (WFPBND) dietary lead-in followed by an 8-week respiratory exchange ratio (RER)-guided walking program.

Randomization was performed using a computer-generated random sequence with equal allocation to LICT, MIIT, and HICT. Group assignments were concealed in sealed opaque envelopes and revealed after completion of baseline testing.

An overview of participant progression through screening, enrollment, allocation, and follow-up is presented in the study flow diagram (Supplementary Material Figure S1).

### 2.2. Participants

Participants were recruited over an 8-week period through church bulletin announcements, flyers, and local business outreach. Of the 66 individuals screened for eligibility, 57 met the inclusion criteria. Six were unable to participate due to scheduling conflicts. Following baseline testing and the 2-week dietary lead-in period, 44 participants entered the intervention. One participant randomized to the high-intensity continuous training (HICT) group withdrew due to inability to meet the required exercise intensity. Forty-four women were randomized; 43 completed the study and were included in analyses.

Women aged 25–54 years with a body mass index (BMI) of 25–35 kg·m^−2^ and a sedentary lifestyle (≤3 exercise sessions·week^−1^; <60 min·week^−1^ of total activity) were recruited. Additional inclusion criteria included body-mass stability for ≥3 months and the ability to walk continuously for 30 min. All participants provided written informed consent prior to participation.

### 2.3. Dietary Intervention

A whole-food, plant-based, non-dairy (WFPBND) diet was implemented beginning two weeks prior to the exercise intervention to promote metabolic stabilization, reduce dietary variability, and attenuate adaptive thermogenesis before metabolic testing. Participants were advised to supplement vitamin B_12_ (500 μg·week^−1^) and vitamin D (600 IU 15 mcg) emphasized by the dietitian per standard whole-food, plant-based, non-dairy (WFPBND) nutritional guidelines. At the end of the lead-in period, resting metabolic rate (RMR) was reassessed via indirect calorimetry, and individualized daily energy prescriptions were derived directly from each participant’s measured RMR. Energy targets were set by subtracting approximately 200 kcal·day^−^^1^; from measured RMR to impose a standardized, modest negative energy balance across participants. Based on individual RMR values, prescribed daily energy intakes ranged from 1000 to 1600 kcal·day^−^^1^.The WFPBND diet emphasized minimally processed plant foods, including vegetables, fruits, whole grains, legumes, and nuts/seeds. Animal products, added oils, refined grains, added sugars, calorically dense processed foods, commercial convenience items, and caffeine were restricted. The intended macronutrient distribution was standardized at approximately 20% protein, 20% fat, and 60% carbohydrate, with dietary fiber intake consistent with whole plant foods. No prescriptive eating window or fasting requirement was imposed; however, participants were encouraged to consume 3-meals/d at regular intervals and to use fiber-rich, low–energy-density foods ad libitum within their assigned caloric limits (~200 kcal·day^−1^ negative energy balance relative to measured RMR).

Dietary adherence was monitored using daily self-reported food logs, which were reviewed weekly by the registered dietitian. Individualized feedback and dietary counseling were provided at each weekly weight-in visit to reinforce compliance and address barriers. Portion-size education and standardized preparation methods consistent with low-oil plant-based eating were emphasized by the dietitian throughout the intervention. Using meal sheets and prepared diet recipes provided by the dietitian gave a structured approach to meal planning that simplified healthy eating and helped consistency and reliability of dietary research goals.

### 2.4. RER-Guided Exercise Intervention

The walking exercise program began in week 3 and continued for 8 weeks at five sessions·week^−1^, progressing in duration by 5 min·week^−1^ from 15 to 50 min·session^−1^. Participants were randomized to one of three metabolic intensities defined by substrate utilization domains:

- Low-Intensity Continuous Training (LICT): Target RER ≈ 0.75 (predominantly fat oxidation).
- Moderate-Intensity Intermittent Training (MIIT): Target RER ≈ 0.85 (balanced fat and carbohydrate oxidation).
- High-Intensity Continuous Training (HICT): Target RER ≈ 0.95 (predominantly carbohydrate oxidation).

Exercise prescriptions were individualized by linking each participant’s measured RER threshold to corresponding heart-rate zones. All exercise sessions were monitored using Polar heart-rate monitors, and intensity adherence was verified daily through computerized logs. This RER-guided framework was chosen to specifically target substrate-utilization domains that differ from traditional VO_2_-based prescriptions, enabling precise metabolic-efficiency training levels [17].

Respiratory exchange ratio (RER; VCO_2_/VO_2_) provides a well-established, non-invasive index of whole-body substrate utilization under steady-state conditions, permitting estimation of the relative contributions of fat and carbohydrate oxidation during rest and exercise [18,19]. This approach is grounded in the seminal crossover concept described by Brooks and Mercier, which characterizes the systematic shift from predominant lipid oxidation toward increasing carbohydrate reliance as exercise intensity increases [19]. Because carbohydrate and fat oxidation during exercise are also influenced by diet and other physiological/environmental factors, individualized prescriptions anchored to measured gas-exchange responses may provide more metabolically specific training targets than workload-only approaches [20]. Within this framework, submaximal threshold concepts provide a physiological basis for identifying transition points in metabolic regulation that are relevant for exercise prescription without requiring maximal-exertion testing [21].

Although respiratory gas exchange is traditionally assessed in laboratory settings, prior work supports the translation of metabolically derived thresholds and individualized physiological markers into field-based exercise prescriptions using heart rate and perceived exertion as practical surrogates [5,17].

### 2.5. Outcome Assessments

Primary outcome measures included changes in body composition, resting metabolic rate and substrate utilization, and cardiorespiratory fitness. All outcome assessments were performed at baseline following the dietary lead-in and repeated after completion of the 8-week exercise intervention.

#### 2.5.1. Anthropometrics and Body Composition

Anthropometric and body-composition measurements were obtained at baseline following the 2-week dietary lead-in and repeated after completion of the 8-week intervention to quantify changes in total body mass, fat mass, lean body mass, and percent body fat.

Body mass, height, and body mass index (BMI) were measured during pre- and post-intervention testing. Body composition (fat mass, fat-free mass, and percent body fat) was assessed using air-displacement plethysmography (BodPod, COSMED USA Inc., Concord, CA, USA) following manufacturer calibration and standardized testing procedures. Air-displacement plethysmography has been widely validated against hydrodensitometry and dual-energy X-ray absorptiometry and is recognized as a reliable method for assessing body composition in adults [22,23,24]. Test–retest reliability for BodPod measurements in our laboratory has demonstrated intraclass correlation coefficients (ICC) > 0.95.

#### 2.5.2. Resting Metabolic Rate

Resting metabolic rate and resting substrate utilization were assessed at baseline following the 2-week dietary lead-in and again after completion of the 8-week exercise intervention to quantify changes in resting energy expenditure and fuel utilization associated with each training condition. Resting metabolic rate was measured using a Parvo Medics TrueOne 2400 metabolic cart (Parvo Medics Inc., Sandy, UT, USA) with a ventilated hood (canopy dilution) system [25]. Measurement procedures were conducted consistently with established hood-based indirect calorimetry methodology and best-practice recommendations for obtaining reliable resting metabolic rate estimates (e.g., standardized pre-test rest and steady-state data selection) [26]. Published validation and reliability evaluations support the use of the Parvo Medics TrueOne 2400 for resting metabolic rate measurement when appropriately calibrated and operated [27]. Participants rested supine in a thermoneutral environment while breath-by-breath VO_2_ and VCO_2_ were collected and averaged to calculate resting energy expenditure. Test–retest reliability for indirect calorimetry in our laboratory has demonstrated intraclass correlation coefficients (ICC) > 0.95.

#### 2.5.3. Graded Metabolic Walking Test

Cardiorespiratory and metabolic responses to progressive walking exercise were evaluated at baseline and post-intervention using a standardized graded treadmill protocol to characterize submaximal gas-exchange responses, including respiratory exchange ratio (RER) behavior and ventilatory/threshold-relevant capacity [17,21,28,29]. VO_2_ and VCO_2_ were measured breath-by-breath using the Parvo Medics TrueOne 2400 metabolic cart, with daily calibration performed using certified calibration gases and a 3 L syringe in accordance with published cardiopulmonary exercise testing (CPET) quality-control recommendations [28,29].

The Southern Adventist University treadmill protocol began at 2.0 mph and increased by 0.5 mph every 3 min; treadmill grade began at 7% and increased by 3% every 3 min, corresponding to approximately 2–3 MET increments per stage [30]. Tests were terminated when RER reached 1.00 to maintain a standardized submaximal metabolic endpoint aligned with the study objective of deriving training-relevant gas-exchange parameters without requiring maximal-exertion criteria [17,28]. Breath-by-breath data were time-aligned and averaged in 15 s intervals, and heart rate was continuously recorded by Polar telemetry [28,29].

### 2.6. Statistical Analysis

A priori sample-size estimate could not be performed by the statistician due to the absence of prior RER-guided walking trials; therefore, a post hoc power analysis was conducted following study completion. All statistical analyses by the statistician were performed using IBM SPSS Statistics (Version 29; IBM Corp., Armonk, NY, USA). Descriptive statistics are presented as means ± standard deviations. Normality, homogeneity of variance, and the presence of outliers were assessed prior to inferential testing. Partial η^2^ values effect sizes were expressed as partial eta squared (η^2^). Based on conventional benchmarks, partial η^2^ values of approximately 0.14, 0.36, and 0.64 were interpreted as small, moderate, and large effects, respectively.

Analysis of covariance (ANCOVA) was used to evaluate post-intervention differences among groups while controlling baseline values. When significant main effects were observed, adjusted post hoc pairwise comparisons were conducted. Effect sizes were reported as partial eta squared (η^2^).

To evaluate within- and between-group changes over time, mixed-factorial ANOVA (group × time) was applied where appropriate. Pearson correlation coefficients were calculated to examine relationships between metabolic variables (e.g., ΔRQ) and body-composition outcomes (e.g., Δ% body fat). Statistical significance was set at *p* < 0.05.

Changes in oxygen uptake at the first ventilatory threshold (VO_2_ at VT1) from baseline to post-intervention were evaluated within and between groups using mixed-factorial ANOVA (group × time). When appropriate, post hoc comparisons were conducted to examine within-group changes over time.

Associations between changes in resting substrate utilization and changes in body composition were examined using Pearson product–moment correlation coefficients, with changes in resting respiratory quotient (ΔRQ) correlated against changes in percent body fat (Δ% body fat). Statistical significance was set at *p* < 0.05.

## 3. Results

### 3.1. Baseline Characteristics

At baseline, no statistically significant differences were observed among LICT, MIIT, and HICT in age, height, body mass, BMI, percent body fat, fat mass, lean body mass, respiratory quotient, or resting energy expenditure (Table 1). Baseline values demonstrated overlapping means and 95% confidence intervals with no between-group differences (*p* > 0.05).

### 3.2. Body Composition

Pre- to post-intervention changes in total body mass, fat mass, lean body mass, percent body fat, and fat-to-lean mass ratio were examined to quantify the effects of the three RER-guided walking intensities under controlled dietary conditions.

Significant time effects were observed for all body-composition variables (ANCOVA time effect, *p* < 0.05), with between-group differences favoring MIIT. MIIT demonstrated the greatest reduction in total body mass (−11.2%), which was significantly greater than LICT (−7.9%) and HICT (−7.2%) (Figure S2, Table 2).

Percent body fat declined most in MIIT (−17.1%) compared with LICT (−12.3%) and HICT (−11.2%) (Figure S3). Absolute fat mass decreased by −25.9% in MIIT, −19.1% in LICT, and −16.7% in HICT (Figure S4).

Changes in lean body mass were small across all groups (Figure S5). The fat-to-lean mass ratio decreased by −24.4% in MIIT, −18.0% in LICT, and −16.3% in HICT (Figure S6). Significant time × treatment interactions were observed for fat mass and percent body fat (*p* < 0.05).

### 3.3. Resting Metabolism and Substrate Utilization

All exercise interventions were associated with significant time effects for resting energy expenditure (REE) (ANCOVA time effect, *p* < 0.0001, η^2^ = 0.492; Table 3). Relative to baseline, REE decreased by 5.6% in LICT, 10.4% in MIIT, and 5.9% in HICT. The largest proportional reduction in REE occurred in the MIIT group (−165 ± 122 kcal·day^−1^ vs. −85 ± 152 kcal·day^−1^ in LICT and −92 ± 147 kcal·day^−1^ in HICT).

Resting energy expenditure declined across all exercise groups. The reduction was greatest in the MIIT group (−165 kcal·day^−1^, −10.4%) compared with LICT (−85 kcal·day^−1^, −5.6%) and HICT (−92 kcal·day^−1^, −5.9%). Parallel reductions were observed in resting oxygen consumption (VO_2_), with MIIT demonstrating the largest decline (−10.1%), followed by HICT (−6.6%) and LICT (−5.0%). Carbon dioxide production (VCO_2_) decreased in all groups, with the largest reduction in MIIT (−12.6%), a moderate reduction in LICT (−8.4%), and minimal change in HICT (−2.7%).

Resting respiratory quotient (RQ) decreased by 3.6% in LICT and 3.0% in MIIT, whereas RQ increased by 4.1% in HICT (*p* = 0.027) (Table 3; Figure S7).

Carbohydrate oxidation (KCHO) declined by 24.1% in LICT and 27.0% in MIIT, but increased by 14.3% in HICT (Figure S8). Change in resting lipid oxidation (KFAT, kcal·day^−^^1^) following 8 weeks of training, showed LICT increased by 18.8%, MIIT increased by 4.0%, whereas HICT decreased by 21.3% from baseline Figure S9). Percent fat oxidation increased by 25.6% in LICT and 15.1% in MIIT, while declining by 15.8% in HICT (Figure S10).

### 3.4. Cardiorespiratory Fitness Outcomes

Cardiorespiratory responses to the three training conditions were evaluated using changes in oxygen uptake at the first ventilatory threshold (VT1) from baseline to post-intervention. VO_2_ at VT1 increased across all groups; however, a statistically significant increase was observed only in the MIIT condition (Δ = 1.487 ± 0.895 L·min^−1^; *p* = 0.038). When expressed relative to body mass, VT1 VO_2_ also increased significantly in the MIIT group (mL·kg^−1^·min^−1^; *p* < 0.05). In the LICT and HICT groups, increases in VO_2_ at VT1 were observed over the intervention period, but these changes did not reach statistical significance (*p* > 0.05).

Changes in resting respiratory quotient (RQ) were positively correlated with changes in percent body fat (r = 0.316, *p* = 0.039).

These coordinated changes in body composition, resting metabolism, and cardiorespiratory fitness provided the basis for the physiological interpretations presented in the Discussion. These outcomes are summarized in Table 3 and Figures S2–S10.

## 4. Discussion

This study evaluated the effects of metabolically guided walking at three distinct respiratory exchange ratio targets on body composition, resting substrate utilization, and submaximal cardiorespiratory fitness in sedentary overweight and obese women. Among the three training conditions, moderate-intensity intermittent training (MIIT) produced the most consistent and integrated improvements across these domains, including greater reductions in adiposity and favorable shifts in resting substrate utilization.

The selective improvement in VO_2_ at the first ventilatory threshold observed in the MIIT group, together with the observed association between resting substrate utilization and adiposity change, suggests coordinated adaptations in submaximal aerobic capacity, resting metabolism, and body composition.

Across all three RER-guided walking prescriptions performed under controlled whole-food, plant-based nutrition and modest energy restriction, participants demonstrated meaningful improvements in adiposity, resting metabolism, and submaximal fitness. The central observation is that walking intensity anchored to measured gas-exchange targets can produce coherent changes in body composition alongside shifts in resting substrate utilization and ventilatory-threshold performance—outcomes that align with the public health need for exercise prescriptions that are both physiologically individualized and practically sustainable [5,17].

A key finding was the divergent resting substrate signatures that emerged across intensity conditions. Two prescriptions were associated with lower resting RQ and higher percent fat oxidation, whereas the high-intensity continuous prescription shifted resting metabolism toward relatively greater carbohydrate reliance. Because resting substrate partitioning is a recognized feature of metabolic flexibility, these patterns are consistent with an intervention effect on the capacity to regulate carbohydrate–fat oxidation in response to energetic demand [9,11,12]. From a mechanistic standpoint, RQ/RER-based outcomes are interpretable within established indirect calorimetry frameworks linking gas exchange to substrate oxidation [18,19], while also acknowledging that diet, training, and physiological context modulate carbohydrate–fat partitioning [20].

Mitochondrial function provides a useful integrative lens for interpreting why a moderate, substrate-targeted “sweet spot” may be advantageous when the goal is fat loss with preserved function. San-Millán emphasizes that mitochondrial oxidative capacity is central to metabolic health and influences substrate selection, metabolic flexibility, and the ability to sustain submaximal workloads with less dependence on carbohydrate [10]. In that context, the combined pattern of improved VT1 and a more favorable resting fuel profile in the moderate-intensity intermittent prescription is consistent with improved oxidative metabolism rather than simply higher external workload—i.e., a phenotype that can support daily activity with greater lipid-supported energy production and improved submaximal performance characteristics [10,17]. Importantly, this interpretation aligns with the study design intent: to derive training-relevant thresholds from submaximal gas-exchange behavior rather than maximal-exertion criteria [17,28,29].

Maintaining lean tissue during weight loss remains clinically important because skeletal muscle supports functional capacity and is a major determinant of resting energy expenditure and metabolic resilience [31,32]. The general preservation of LBM across conditions in this trial is therefore meaningful in its own right, and it strengthens the practical relevance of the weight-loss response under modest energy restriction [26,27]. Within a public-health frame, an approach that reduces adiposity while limiting lean-mass loss is particularly appealing because it supports mobility, reduces deconditioning risk, and improves the likelihood that participants can continue exercising after a structured program ends [26,27].

The less favorable resting fuel pattern observed with continuous high-intensity training under energy restriction also deserves careful interpretation. High-intensity approaches can be effective for weight reduction, but they may be harder to sustain for previously sedentary individuals, and long-term adherence advantages are not guaranteed [8]. When paired with an energy deficit, a more carbohydrate-dominant resting profile may reflect a physiological state that is not optimized for lipid-supported daily living. One plausible explanation is that higher-intensity continuous work relies more heavily on glycolytic flux during training bouts, potentially influencing downstream substrate handling at rest, whereas repeated exposure to moderate oxidative workloads may better support lipid-oxidation capacity and flexible substrate switching [9,10,20]. These are mechanistic inferences (not directly measured at the muscle level here), but they are consistent with the observed directionality of RQ/RER outcomes and the metabolic-flexibility construct [9,11,12].

The dietary context likely amplified the translational value of the findings. Whole-food, plant-based dietary patterns are associated with improved cardiometabolic risk profiles and cardiovascular health, and recent syntheses suggest such patterns can support aerobic performance outcomes without compromising strength or power performance when appropriately planned [13,14,15,16]. In this study, applying a standardized, modest negative energy balance using measured RMR helped reduce variability from dietary intake and strengthened attribution of between-condition differences to the exercise-intensity domain rather than to uncontrolled dietary fluctuations. Within this controlled nutritional backdrop, the prescription that produced the most integrated outcome profile—combining adiposity reductions with favorable resting substrate shifts and a significant VT1 improvement—appears particularly promising as a feasible public-health strategy because it relies on walking rather than specialized equipment or advanced athletic skill.

Finally, the present results reinforce a practical limitation of traditional “one-size-fits-all” intensity prescriptions: individuals can show different metabolic responses at the same relative workload, and externally imposed targets (e.g., %HRmax or %VO_2_max) may not consistently place participants in the same substrate-utilization domain [5]. By contrast, using laboratory-derived RER thresholds and translating them into individualized heart-rate zones offers a path to preserve personalization while improving real-world scalability [5,17]. If future work confirms durability and adherence, a simple workflow—brief gas-exchange assessment, individualized heart-rate zones mapped to substrate targets, and walking-based training—could help community programs prescribe intensities that are both tolerable and metabolically meaningful.

In future research, the following study’s methodological constraints outlined below should be considered.

### 4.1. Integrated Interpretation of Percent Changes in Resting Metabolism

Although all three exercise prescriptions produced time-dependent reductions in resting energy expenditure (−5.6% to −10.4%), the magnitude and direction of substrate utilization differed by training intensity. LICT and MIIT exhibited coordinated reductions in resting respiratory quotient, substantial suppression of carbohydrate oxidation (−24.1% to −27.0%), and increases in percent fat oxidation (+15.1% to +25.6%). In contrast, HICT demonstrated increases in respiratory quotient (+4.1%), elevated carbohydrate oxidation (+14.3%), and reductions in percent fat oxidation (−15.8%). These divergent percent-based responses indicate intensity-specific modulation of resting fuel utilization and provide a mechanistic context for the observed between-group differences in body-composition outcomes.

### 4.2. Practical Interpretation

Under conditions of controlled whole-food, plant-based nutrition and modest energy restriction, all three RER-guided walking prescriptions produced meaningful reductions in body mass and fat mass. However, the qualitative pattern of resting substrate utilization differed by training intensity. Participants in LICT and MIIT demonstrated reductions in respiratory quotient and carbohydrate oxidation with concomitant increases in percent fat oxidation, whereas HICT shifted resting metabolism toward greater carbohydrate reliance.

From a practical standpoint, a lower resting respiratory quotient and higher percent fat oxidation reflect a metabolic phenotype that preferentially utilizes stored lipid as a primary fuel source during daily living. The resting metabolic profile observed with MIIT appears particularly advantageous, as it combines the largest absolute fat loss with improved resting fuel efficiency and significant enhancement in ventilatory threshold performance.

These findings suggest that prescribing walking intensity within a moderate, substrate-optimized metabolic domain may facilitate more sustainable fat loss while preserving lean tissue and improving aerobic capacity. In contrast, continuous high-intensity exercise, although effective for weight reduction, may promote a resting fuel profile less favorable for long-term fat loss under caloric restriction.

A conceptual graphical abstract is provided to summarize the observed relationships among exercise intensity, respiratory exchange ratio (RER), and integrated metabolic outcomes, illustrating that moderate, metabolically guided effort was associated with the most favorable overall pattern of response.

### 4.3. Limitations

This study has several limitations that should be considered when interpreting the findings. First, the modest sample size resulted in moderate achieved statistical power; however, post hoc analyses based on changes in resting energy expenditure demonstrated moderate effect sizes for MIIT versus LICT (d = 0.58) and MIIT versus HICT (d = 0.54), with achieved power of approximately 0.55–0.60, supporting the physiological relevance of the observed between-group metabolic differences and the feasibility of larger trials.

Second, the sample included only sedentary, overweight, and obese middle-aged women, which limits generalizability to men, younger or older adults, and individuals with different health or fitness profiles. Third, the intervention duration (2-week dietary lead-in followed by 8 weeks of exercise) was sufficient to detect significant physiological and metabolic adaptations, but does not permit conclusions regarding long-term adherence, durability of adaptations, or weight-maintenance outcomes. Fourth, although the whole-food, plant-based dietary protocol was structured with prescribed menus and monitored weekly using standardized daily dietary check sheets, dietary intake was self-reported and therefore subject to reporting bias. Finally, the absence of a non-exercise control group limits complete isolation of the independent effects of exercise prescription from dietary change alone.

It should also be noted that the study was designed to evaluate metabolic efficiency and substrate utilization at submaximal intensities rather than maximal aerobic performance. Accordingly, metabolic testing was terminated at a respiratory exchange ratio (RER) of 1.0 rather than the conventional RER ≥ 1.15 used to validate maximal oxygen uptake. This submaximal threshold-based testing enhanced participant safety while aligning directly with the study objective of identifying training intensities that most effectively shift substrate utilization toward greater fat oxidation.

Despite these limitations, the consistency, magnitude, and physiological coherence of the observed metabolic and body-composition adaptations across exercise intensities support the translational relevance of metabolically guided walking combined with plant-based nutrition. These findings provide a rationale for larger and longer-term randomized trials across more diverse populations.

### 4.4. Implications for Practice

Given the low barrier and broad reach of walking-based interventions, the present findings highlight the potential relevance of translating metabolically guided intensity targets into heart-rate–based frameworks for population-level contexts.

Determining metabolically efficient exercise intensities typically requires access to laboratory-based metabolic testing. Based on the present findings, heart-rate zones corresponding to a respiratory exchange ratio of approximately 0.80–0.85 may provide a practical proxy for intensities that favor fat oxidation in sedentary, overweight adults.

To support translation, a heart-rate calculator based on the RER-guided framework was developed and is available at https://www.smhearter.com/ (accessed on 18 January 2026). The tool provides a pragmatic approximation of substrate-targeted heart-rate zones when metabolic testing is unavailable (see Supplementary File S11). Calculator estimates were not used in this study; all prescriptions and analyses were derived from laboratory-based metabolic testing.

In addition, the whole-food, plant-based research diet, including menus and recipes, can be obtained by contacting the research dietitian (Dorothea Sarli) at dsarli@southern.edu or via https://www.sarlinutrition.com/ (accessed on 18 January 2026).

These resources may assist clinicians, practitioners, and community programs in implementing metabolically guided walking and plant-based nutrition strategies in real-world settings. Users should consider individual health status, preferences, and local practice guidelines when applying these tools.

Collectively, these findings indicate that metabolically guided, moderate-intensity intermittent walking produces a favorable pattern of body composition, resting substrate utilization, and submaximal aerobic adaptations when performed under standardized whole-food, plant-based nutritional conditions. By targeting individualized fuel-utilization domains rather than conventional percentage-based intensity prescriptions, this approach may represent a physiologically relevant and practically sustainable strategy for obesity intervention.

Future studies should examine longer-term adherence and should compare RER-guided exercise directly with traditional percentage-based intensity prescriptions including pragmatic community-implementation trials.

## 5. Conclusions

This study demonstrates that combining metabolically guided moderate-intensity intermittent walking with a controlled whole-food, plant-based diet and a modest negative energy balance produces meaningful improvements in body composition, resting substrate utilization, and submaximal cardiorespiratory fitness in sedentary overweight or obese adult women. Among the three exercise intensities tested, moderate-intensity intermittent training elicited the most favorable and integrated outcomes, including a mean reduction in percent body fat of −6.6 ± 2.2% with relative preservation of lean body mass (−0.82 ± 1.38 kg). These adaptations were accompanied by increased resting fat oxidation, improved fat-to-lean mass ratio, and enhanced submaximal aerobic performance, consistent with improved metabolic flexibility.

Collectively, the findings identify a practical metabolic “sweet spot” for exercise prescription—corresponding to approximately two-thirds fat and one-third carbohydrate utilization—that appears physiologically effective while remaining tolerable for previously sedentary individuals. From a public-health perspective, these results support the feasibility of translating fuel-informed walking prescriptions into community and clinical contexts without reliance on maximal testing or complex intensity targets.

Future research should examine the long-term sustainability of RER-guided exercise, including adherence, weight-maintenance outcomes, and cardiometabolic health endpoints, and should directly compare metabolically guided prescriptions with traditional percentage-based intensity approaches using both laboratory-based and pragmatic field implementations.

## Figures and Tables

**Table 1:** Anthropometric and Body Composition Characteristics.

	LICT (n = 14)	MIIT (n = 15)	HICT (n = 14)
Age (y)	43 ± 8	42 ± 7	43 ± 7
Height (cm)	163 ± 6	164 ± 8	168 ± 9
BMI (m × kg^−2^)	30.6 ± 5.5	29.1 ± 5.9	28.4 ± 5.0
Total Body Mass (kg)	83.0 ± 15.7	78.6 ± 17.4	80.3 ± 15.8
Fat Mass (kg)	32.5 ± 12	30.9 ± 11	31.7 ± 12
Lean Body Mass (kg)	49.3 ± 5.5	47.7 ± 8.7	48.6 ± 5.9
%-Fat Mass	39.7 ± 5.4	38.7 ± 6.0	38.4 ± 7.7
Respiratory Quotient (VCO_2_·VO_2_^−1^)	0.87 ± 0.08	0.84 ± 0.08	0.83 ± 0.06
Resting Energy Expenditure (kcal·day^−1^)	1527 ± 209	1580 ± 256	1564 ± 258

Data are presented as mean ± SD [95% CI]. Between-group differences were assessed using one-way ANOVA. BMI, LBM, %FM, RQ, REE.

**Table 2:** Treatment effects (8 weeks) of (i) low-intensity, continuous training (LICT, n = 14), (ii) moderate-intensity, intermittent training (MIIT, n = 15), or (iii) high-intensity, continuous training (HICT, n = 14), combined with a 10-week controlled whole-food, plant-based diet and a 200 kcal·day^−1^ caloric restriction on body composition parameters.

Variable	LICT Pre	LICT Post	∆	MIIT Pre	MIIP Post	∆	HICT Pre	HICT Post	∆	ANCOVA, *p*-Value [η^2^_p_ Effect Size] (Treatment, Time, Interaction)
BMI, kg·m^−2^	30.6 ± 5.5 [27.5–33.6]	28.1 ± 4.9 ** [25.4–30.8]	−2.4 ± 1.1 ^‡^ [−3.0–−1.8]	29.1 ± 5.9 [25.8–32.3]	25.9 ± 5.5 ** [22.8–28.9]	−3.2 ± 0.6 ^†^ [−3.5–−2.9]	28.4 ± 5.0 [25.6–31.3]	26.3 ± 4.6 ** [23.7–29.0]	−2.1 ± 0.9 ^‡^ [−2.6–−1.5]	(0.001 [0.314], 0.000 [0.926], 0.000 [0.286])
Total Body Mass, kg	81.8 ± 16 [73.0–90.6]	75.2 ± 14 ** [67.6–83.0]	−6.5 ± 3.1 ^‡^ [−8.3–−4.8]	78.6 ± 17 [69.0–88.2]	69.9 ± 16 ** [61.0–78.7]	−8.8 ± 1.9 ^†^ [−9.9–−7.7]	8.3 ± 16 [71.2–89.5]	74.5 ± 15 ** [65.8–83.2]	−5.8 ± 2.5 ^‡^ [−7.2–−4.4]	(0.001 [0.314], 0.000 [0.925], 0.000 [0.338])
Fat Mass, kg	32.5 ± 12 [26.0–39.0]	26.4 ± 10 ** [20.7–32.1]	−6.2 ± 2.9 [−7.8–−4.6]	30.9 ± 11 [25.0–36.8]	23.0 ± 9.3 ** [17.8–28.1]	−8.0 ± 2.3 [−9.3–−6.6]	31.7 ± 12 [24.5–38.9]	26.3 ± 12 ** [19.4–33.2]	−5.3 ± 2.9 ^‡^ [−7.1–−3.7]	(0.012 [0.198], 0.000 [0.892], 0.001 [0.249])
Lean Body Mass, kg	49.3 ± 5.5 [46.2–52.4]	48.9 ± 5.3 [46.0–51.8]	−0.38 ± 2.06 [−1.52–0.77]	47.7 ± 8.7 [42.9–52.5]	46.9 ± 8.5 * [42.2–51.6]	−0.82 ± 1.38 [−1.59–−0.06]	48.6 ± 5.9 [45.3–52.0]	48.2 ± 6.1 [44.7–51.8]	−0.40 ± 1.65 [−1.36–0.55]	(––, 0.046 [0.096], ––)
Body Fat, %	38.9 ± 6.1 [35.3–42.3]	34.1 ± 6.9 ** [30.3–38.0]	−4.8 ± 2.7 [−6.3–−3.3]	38.7 ± 6.0 [35.3–42.0]	32.1 ± 6.6 ** [28.4–35.7]	−6.6 ± 2.2 [−7.8–−5.4]	38.4 ± 7.7 [33.9–42.8]	34.1 ± 8.4 ** [29.2–39.0]	−4.3 ± 2.6 ^‡^ [−5.8–−2.8]	(0.039 [0.150], 0.000 0.827], ––)

Data are presented as mean ± SD [95% CI]. Two-way repeated-measures ANCOVA with baseline as covariate; Bonferroni-adjusted post hoc tests. *, ** *p* < 0.05, *p* < 0.01 vs. pre-intervention; † *p* < 0.05 vs. LICT; ‡ *p* < 0.05 vs. MIIT.

**Table 3:** Treatment effects (8 weeks) of (i) low-intensity, continuous training (LICT, n = 14), (ii) moderate-intensity, intermittent training (MIIT, n = 15), or (iii) high-intensity continuous training (HICT, n = 14), combined with 10-week controlled whole-food, plant-based diet and a 200-kcal·day^−1^ caloric restriction on resting parameters.

Variable	LICT Pre	LICT Post	∆	MIIT Pre	MIIT Post	∆	HICT Pre	HICT Post	∆	ANCOVA, *p*-Value [η^2^_p_ Effect Size] (Treatment, Time, Interaction)
Respiratory Quotient, VCO_2_/VO_2_	0.87 ± 0.08 [0.82–0.91]	0.84 ± 0.04 [0.81–0.86]	−0.031 ± 0.08 [−0.07–0.01]	0.84 ± 0.08 [0.80–0.89]	0.82 ± 0.06 * [0.78–0.85]	−0.025 ± 0.05 [−0.05–0.00]	0.83 ± 0.06 [0.79–0.86]	0.86 ± 0.05 * [0.83–0.89]	−0.034 ± 0.06 ^‡†^ [0.00–0.07]	(0.006 [0.228], ––, 0.000 [0.519])
VO_2_, mL·min^−1^	218 ± 31 [201–235]	208 ± 28 [192–223]	−11 ± 21 [−22–1]	227 ± 37 [207–248]	205 ± 32 ** [187–223]	−23 ± 18 [−32–−13)]	226 ± 36 [205–247]	211 ± 30 * [194–228]	−15 ± 21 [−27–−3)]	(––, 0.000 [0.489], 0.001 [0.261])
VCO_2_, mL·min^−1^	190 ± 27 [175–205]	174 ± 20 * [162–185]	−16 ± 226 [−30–(−2]	191 ± 34 [172–210]	167 ± 27 ** [152–182]	−24 ± 17 [−34–−15)]	187 ± 31 [169–205]	182 ± 27 [166–197]	−5 ± 21 ^‡^ [−17–7]	(0.041 [0.148], 0.000 [0.454], 0.000 [0.367])
Energy Expenditure, kcal·day^−1^	1527 ± 209 [1411–1642]	1442 ± 185 * [1339– 1544]	−85 ± 152 [−169–(−1]	1580 ± 256 [1438– 1722]	1415 ± 218 ** [1293– 1536]	−165 ± 122 [−233–−98)]	1564 ± 248 [1421– 1707]	1472 ± 208 * [1352– 1592]	−92 ± 147 [−177–−8)]	(––, 0.000 [0.492], 0.000 [0.271])
K_CHO_ Oxidation Rate, kcal·day^−1^	875 ± 398 [654–1095]	664 ± 204 [551–776]	−211 ± 393 [−429–7]	748 ± 477 [484–1013]	547 ± 311 * [375–719]	−202 ± 2.84 [−359–−45)]	680 ± 322 [494–865]	776 ± 268 [621–931]	97 ± 322 ^‡^ [−90–283]	(0.011 [0.201], 0.003 [0.203], 0.000 [0.579])
K_FAT_ Oxidation Rate, kcal·day^−1^	660 ± 422 [426–894]	784 ± 278 [630–938]	124 ± 325 [−56–304]	840 ± 481 [573–1106]	874 ± 345 [682–1065]	34 ± 284 [−123–191]	892 ± 345 [693–1091]	702 ± 265 * [548–855]	−190 ± 309 [−369–−12)]	(0.033 [0.157], ––, 0.000 [0.497])
K_FAT_ Oxidation, %	43 ± 26 [29–58]	54 ± 14 [46–62]	11 ± 25 [−3–25]	53 ± 27 [39–68]	61 ± 22 [49–73]	8 ± 16 [−1–17]	57 ± 20 [45–69]	48 ± 16 [38–57]	−9 ± 21 ^‡^ [−21–3]	(0.027 [0.164], ––, 0.000 [0.504])

Data are presented as mean ± SD [95% CI]. Two-way repeated-measures ANCOVA with baseline as covariate; Bonferroni-adjusted post hoc tests. *, ** *p* < 0.05, *p* < 0.01 vs. pre-intervention; † *p* < 0.05 vs. LICT; ‡ *p* < 0.05 vs. MIIT.

## Data Availability

De-identified data and analytic code (descriptive statistics, ANCOVA, and general linear modeling outputs) are available in Excel format via the Southern Adventist University McKee Library Knowledge Exchange at: https://knowledge.e.southern.edu/datasets/1/ (accessed on 18 January 2026).

## Supplementary Materials

The following supporting information can be downloaded at: https://www.mdpi.com/article/10.3390/ijerph23010136/s1, Figure S1: Study flow diagram; Figure S2: Change in total body mass after 8 weeks in LICT, MIIT, and HICT; Figure S3: Change in percent body fat after 8 weeks in LICT, MIIT, and HICT; Figure S4: Change in fat mass after 8 weeks in LICT, MIIT, and HICT; Figure S5: Change in lean body mass after 8 weeks in LICT, MIIT, and HICT; Figure S6: Change in fat-to-lean body mass ratio after 8 weeks in LICT, MIIT, and HICT; Figure S7: Change in resting respiratory quotient (RQ) after 8 weeks in LICT, MIIT, and HICT; Figure S8: Change in resting carbohydrate oxidation (KCHO, kcal·day^−^^1^) after 8 weeks in LICT, MIIT, and HICT; Figure S9: Change in resting lipid oxidation (KFAT, kcal·day^−^^1^) following 8 weeks of training; Figure S10. Change in resting percent fat oxidation after 8 weeks in LICT, MIIT, and HICT; File S11: Design of Sweet Spot Heart Rate Calculator (SS_EHR).
