# Supplementary material for: Metabolically Guided Walking and Plant-Based Nutrition Enhance Body Composition and Weight Loss
Source: Int J Environ Res Public Health. 2026 Jan 22;23(1):136. doi: 10.3390/ijerph23010136 (PMC12840797; doi:10.3390/ijerph23010136)
Supplement: Supplementary file 1 [file ijerph-23-00136-s001.zip › ijerph-3879413-supplementary.pdf]

Supplementary Materials

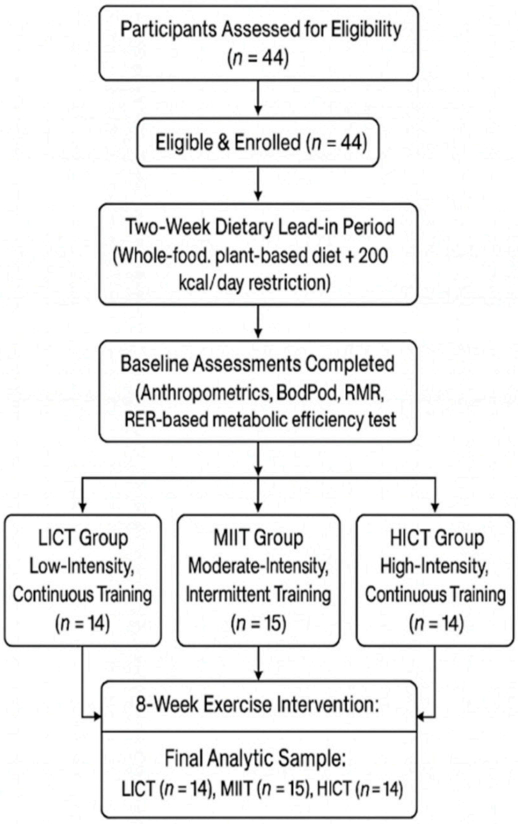

Figure S1. Study flow diagram. Forty-four participants entered the intervention following consent and enrollment. One participant randomized to high-intensity continuous training (HICT) withdrew during the intervention due to inability to meet the prescribed exercise intensity. The final analytic sample included 43 participants.

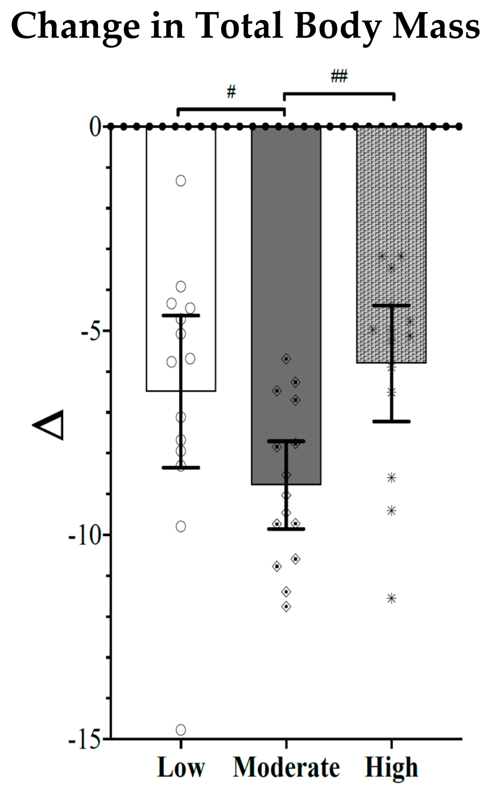

Figure S2. Change in total body mass after 8 weeks in LICT, MIIT, and HICT. Total body mass decreased by **7.9%** in LICT, **11.2%** in MIIT, and **7.2%** in HICT from baseline. Data are presented as mean ± SD with 95% confidence intervals. †$p < 0.05$ vs. LICT; ‡$p < 0.05$ vs. MIIT.

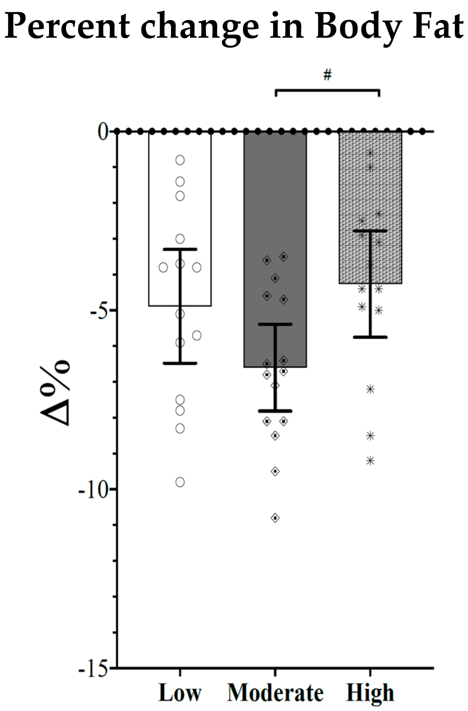

Figure S3. Change in percent body fat after 8 weeks in LICT, MIIT, and HICT. Percent body fat decreased by **12.3%** in LICT, **17.1%** in MIIT, and **11.2%** in HICT from baseline. Data are presented as mean ± SD with 95% confidence intervals. †*p* < 0.05 vs. LICT; ‡*p* < 0.05 vs. MIIT.

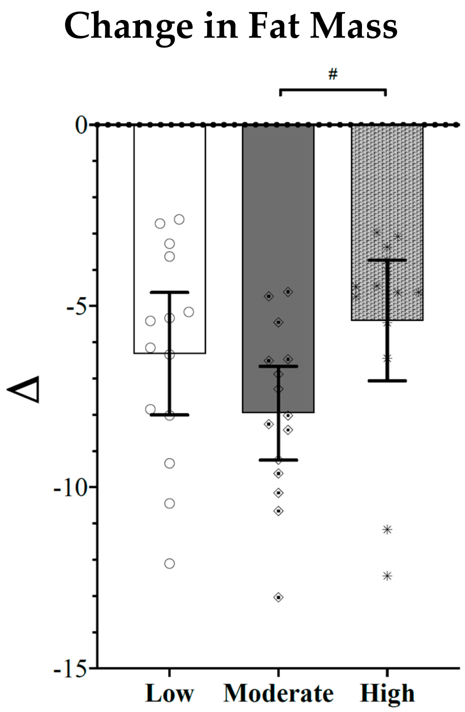

Figure S4. Change in fat mass after 8 weeks in LICT, MIIT, and HICT. Fat mass decreased by **19.1%** in LICT, **25.9%** in MIIT, and **16.7%** in HICT from baseline. Data are presented as mean ± SD with 95% confidence intervals. †$p < 0.05$ vs. LICT; ‡$p < 0.05$ vs. MIIT.

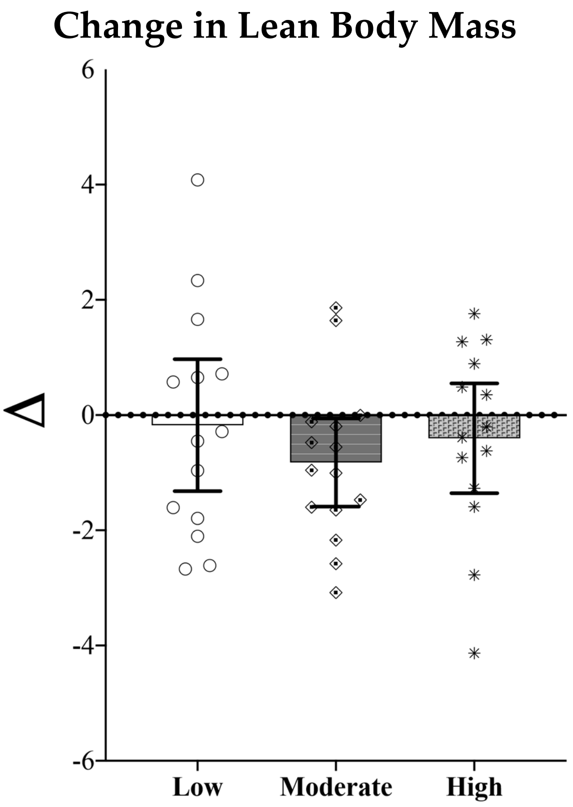

Figure S5. Change in lean body mass after 8 weeks in LICT, MIIT, and HICT. Lean body mass changed by **−0.2%** in LICT, **−1.7%** in MIIT, and **−0.8%** in HICT from baseline. Data are presented as mean ± SD with 95% confidence intervals. †$p < 0.05$ vs. LICT; ‡$p < 0.05$ vs. MIIT.

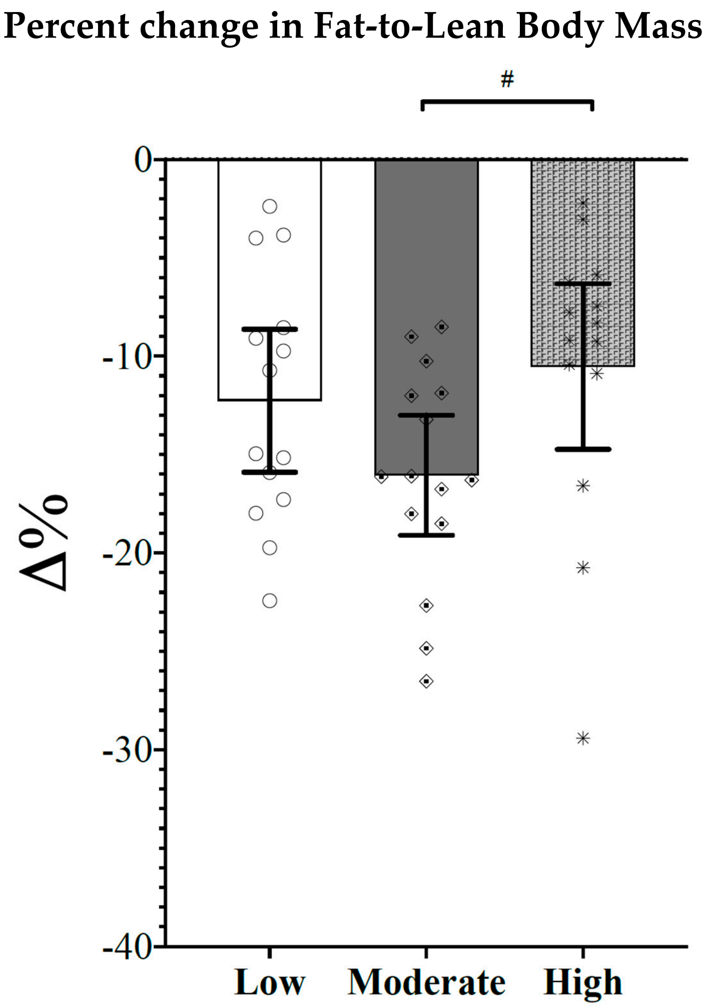

Figure S6. Change in fat-to-lean body mass ratio after 8 weeks in LICT, MIIT, and HICT. Fat-to-lean ratio decreased by **18.0%** in LICT, **24.4%** in MIIT, and **16.3%** in HICT from baseline. Data are presented as mean ± SD with 95% confidence intervals. †$p < 0.05$ vs. LICT; ‡$p < 0.05$ vs. MIIT.

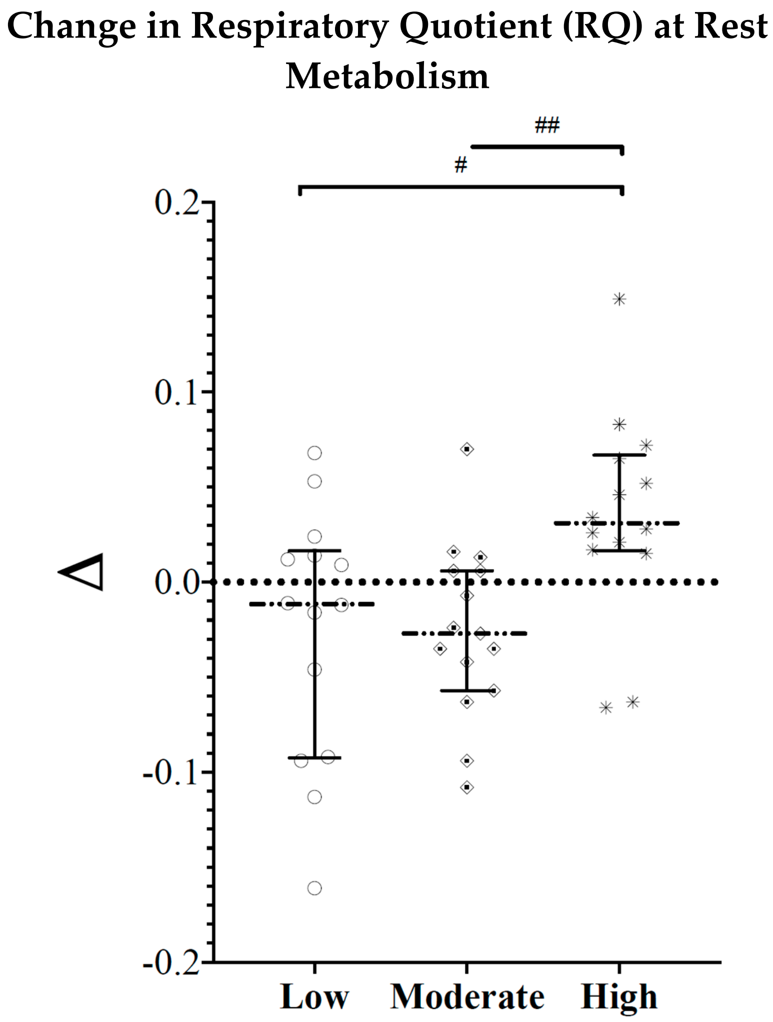

Figure S7. Change in resting respiratory quotient (RQ) after 8 weeks in LICT, MIIT, and HICT. RQ decreased by 3.6% in LICT, decreased by 3.0% in MIIT, and increased by 4.1% in HICT. Data are presented as mean ± SD with 95% confidence intervals.

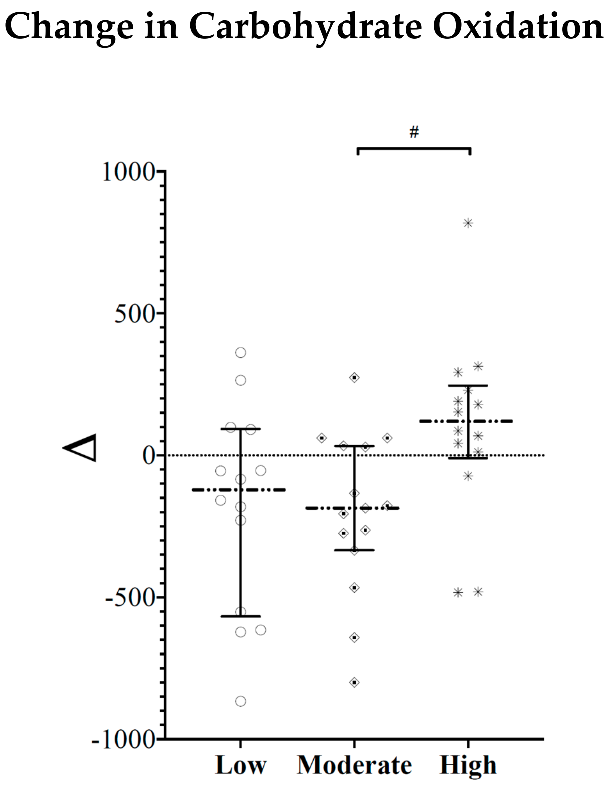

Figure S8. Change in resting carbohydrate oxidation (KCHO, kcal·day^{-1}) after 8 weeks in LICT, MIIT, and HICT. LICT decreased by 24.1%, MIIT decreased by 27.0%, and HICT increased by 14.3% from baseline. Data are presented as mean ± SD with 95% confidence intervals. †$p < 0.05$ vs. LICT; ‡$p < 0.05$ vs. MIIT.

Change in Substrate Fat Oxidation Rates

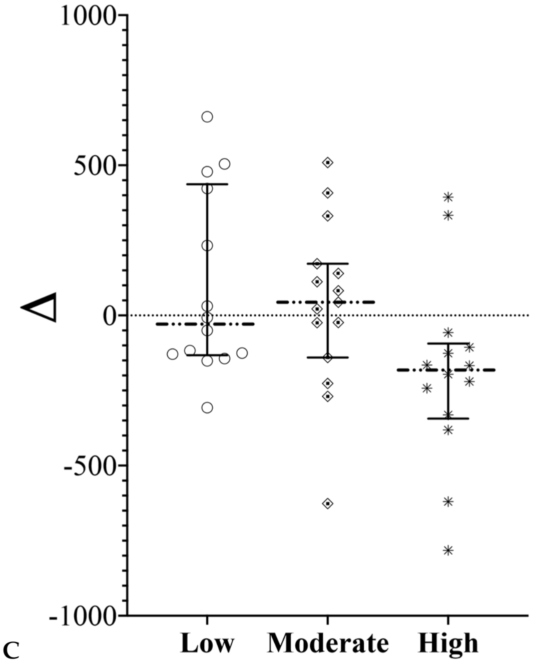

Figure S9. Change in resting lipid oxidation (KFAT, kcal·day^{-1}) following 8 weeks of training. LICT increased by **18.8%**, MIIT increased by **4.0%**, whereas HICT decreased by **21.3%** from baseline. Data are presented as mean ± SD with 95% confidence intervals. †$p < 0.05$ vs. LICT; ‡$p < 0.05$ vs. MIIT.

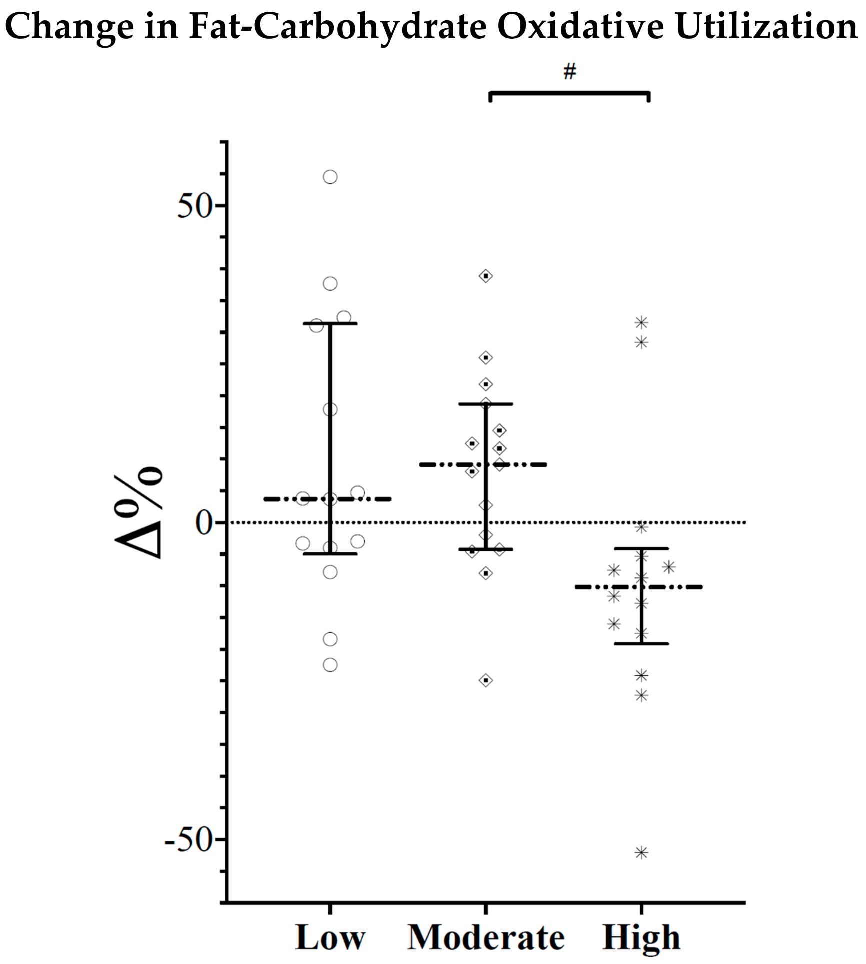

Figure S10. Change in resting percent fat oxidation after 8 weeks in LICT, MIIT, and HICT. Percent fat oxidation increased by 25.6% in LICT, increased by 15.1% in MIIT, and decreased by 15.8% in HICT. Data are presented as mean ± SD with 95% confidence intervals. †$p < 0.05$ vs. LICT; ‡$p < 0.05$ vs. MIIT.

File S11. Design of Sweet Spot Heart Rate Calculator (SS_EHR)

A proof-of-concept linear regression model was developed to estimate sweet-spot exercise heart rate (SS_EHR)—i.e., the exercise heart-rate zone intended to approximate the substrate-utilization "sweet spot" targeted in this trial—using routinely available inputs (age, sex, height, body mass, and resting heart rate). De-identified laboratory records (n = 380) from a university human performance laboratory were split into training (83%) and independent test (17%) subsets. On the held-out test set, model performance showed moderate explanatory power ($R^2 = 0.31$) with mean squared error (MSE) = 219.75 bpm^2 (RMSE ≈ 14.8 bpm). Visual inspection of observed-versus-predicted values indicated similar error structure across training and test sets without evidence of overfitting, while residual dispersion suggests that additional predictors (e.g., fitness indices and autonomic markers) will be needed to improve precision in future iterations. Accordingly, the calculator is intended as a low-burden approximation tool for community and prevention settings when metabolic testing is not available, rather than a replacement for individualized laboratory assessment or clinical evaluation.

Design and programming: Anjan Sapkota, MSCS, Southern Adventist University.
